# An Immigrant With Intestinal Myiasis in the United States: A Case Report and a Review of the Literature

**DOI:** 10.7759/cureus.102716

**Published:** 2026-01-31

**Authors:** Faris Shweikeh, Jad Kabbara, Colin Fricker, Mohamad Mouchli

**Affiliations:** 1 Internal Medicine, Cleveland Clinic Akron General, Akron, USA; 2 Anesthesiology, Lake Erie College of Osteopathic Medicine, Erie, USA; 3 Internal Medicine, Northeast Ohio Medical University, Rootstown, USA; 4 Gastroenterology, Fischer-Titus Medical Center, Westlake, USA

**Keywords:** endoscopy, gastroenterology, insect larvae, intestinal myiasis, parasites

## Abstract

Myiasis is an infestation of human tissue by fly larvae. Intestinal myiasis occurs when fly eggs or larvae in contaminated food or water survive passage through the gastrointestinal tract and are found in the stool or bowel lumen. It is more prevalent in developing countries and can present with nonspecific gastrointestinal symptoms that mimic common conditions.

A 55-year-old Nepalese-American woman, with a long history of intermittent abdominal pain, nausea, and vomiting, underwent CT imaging, which demonstrated a 2 cm colonic narrowing with wall thickening of the proximal transverse colon. Colonoscopy revealed larvae in the terminal ileum and transverse colon, with adjacent inflammation, and biopsy showed eosinophilic infiltration. Microbiology identified eggs that could not be speciated, and a working diagnosis of intestinal myiasis was made. The patient received bowel preparation and washout, with infectious disease follow-up.

Diagnosis was established by direct endoscopic visualization and biopsy. Management focused on mechanical washout, supportive care, and counseling regarding food safety and sanitation.

Intestinal myiasis should be considered in patients with unexplained chronic gastrointestinal symptoms, especially those with dietary exposure to food from endemic regions. Direct visualization during endoscopy simplifies diagnosis. Recognition of myiasis may prevent unnecessary prolonged workup and guide appropriate, noninvasive treatment.

## Introduction

Myiasis is defined as an infestation of human tissue by fly larvae, a phenomenon that commonly involves the skin or natural orifices and is prevalent in tropical regions [1]. There are multiple presentations of myiasis, with the most common being cutaneous myiasis [2]. However, a particularly rare form is intestinal myiasis, which occurs when fly eggs or larvae present in food or water are ingested and survive passage through the gastrointestinal tract.

As stomach acidity is a harsh environment for many pathogens, larvae that cannot endure gastric acidity and perish are termed pseudomyiasis. Due to the harsh nature of the gastrointestinal environment and the unsuitability of humans as a host, true intestinal myiasis is usually accidental; therefore, it is rare for flies to have an ongoing life cycle within the gut mucosa [2]. Clinical manifestations of intestinal myiasis are variable: patients may be entirely asymptomatic, or may have nonspecific gastrointestinal complaints such as abdominal pain, nausea, vomiting, diarrhea, or even rectal bleeding [3]. Because symptoms often mimic other conditions, diagnosis is rarely suspected until larvae are observed in the stool or during endoscopic evaluation.

Fly species implicated in intestinal myiasis span several families, with causative genera including *Musca*, *Sarcophaga*, *Lucilia*, *Eristalis*, *Hermetia*, and drain flies [2-4]. Notably, most documented cases occur in individuals from endemic regions with exposure to contaminated food and water, making intestinal myiasis in the United States a diagnosis that can be easily overlooked. To our knowledge, only 13 cases were reported in developed countries between 2000 and 2019. Therefore, to our knowledge, we describe the first reported case in the United States of intestinal myiasis in a Nepalese-American patient with chronic gastrointestinal symptoms, with a discussion of the relevant literature.

This article was previously presented as a meeting abstract at the 2024 ACG EPoster conference on October 28, 2024.

## Case presentation

A 55-year-old Nepalese woman who immigrated to the United States over a decade ago, with a past medical history of multinodular thyroid disease and latent tuberculosis, had undergone an extensive workup for chronic intermittent abdominal pain, nausea, and vomiting five years prior, which was inconclusive. She was taking linaclotide, hyoscyamine, docusate sodium, polyethylene glycol, and omeprazole. Her diet consisted of well-cooked meat, rice, yogurt, and vegetables, and she often shopped at a local Nepalese grocery store. Her water at home was purified city water.

**Figure 1:**
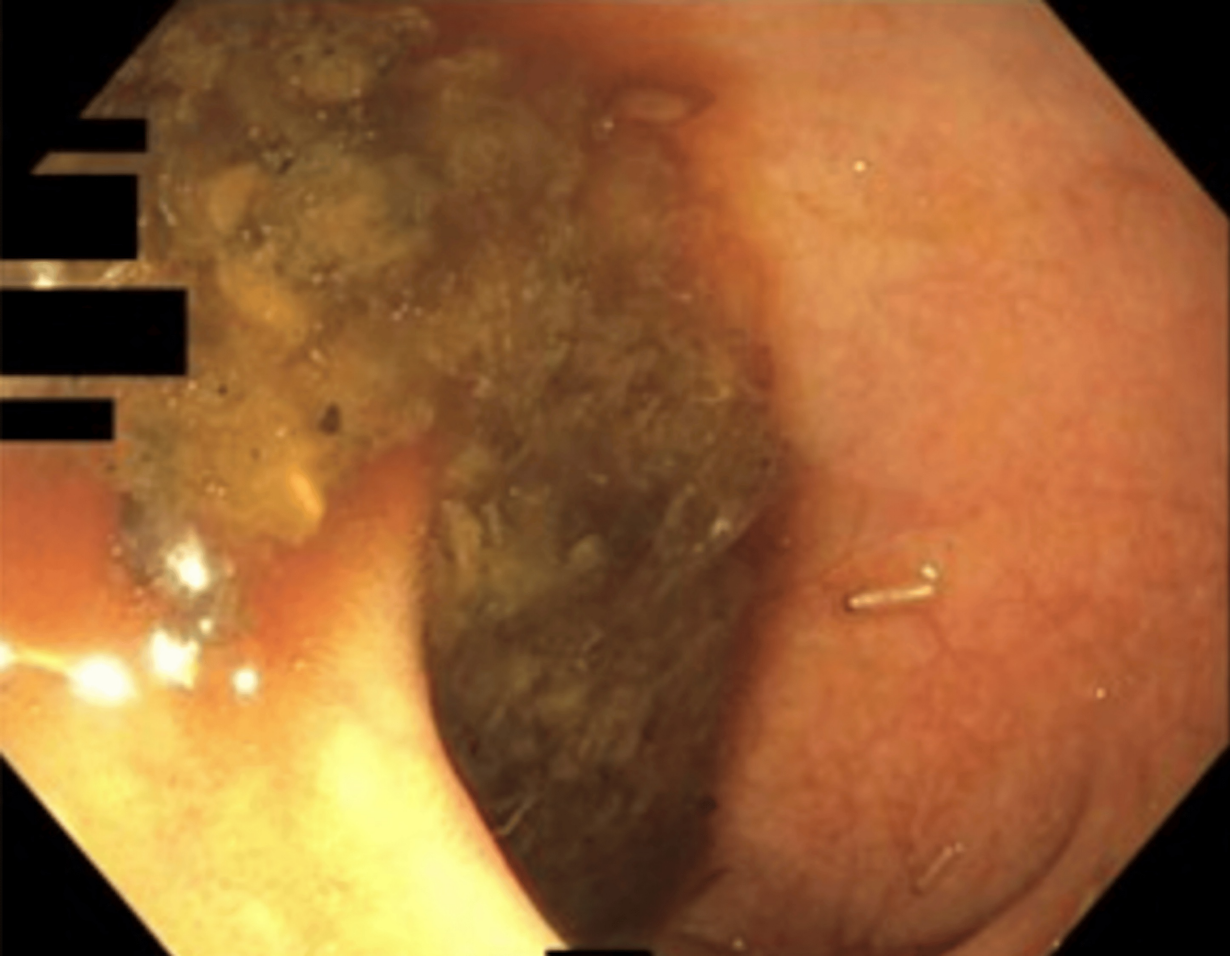
Low-power endoscopic view of the proximal transverse colon, showing luminal debris and a single translucent, segmented larva adherent to inflamed mucosa.

**Figure 2:**
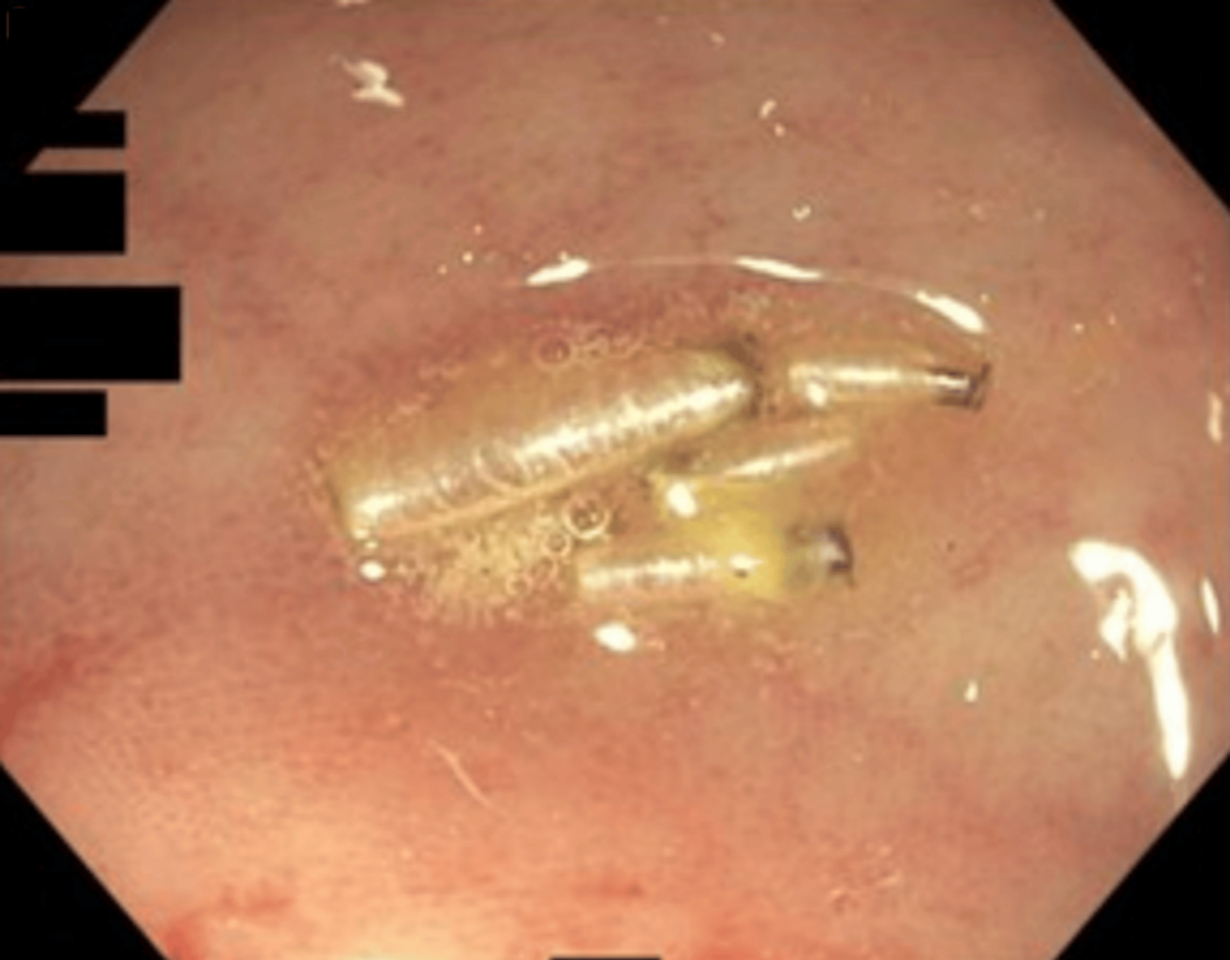
Higher-magnification endoscopic close-up demonstrating multiple live, translucent, segmented larvae attached to the mucosal surface, with adjacent erythema. A biopsy taken from the nearby mucosa demonstrated eosinophilic infiltration.

After presenting to the Emergency Department for worsening symptoms, she was directed to follow up with her primary care physician and gastroenterologist. CT imaging showed a 2 cm colonic narrowing and wall thickening in the proximal transverse colon. On colonoscopy, larvae of an unknown parasite were found in the terminal ileum and transverse colon (Figures 1-2), along with inflammation and a stricture. She had three previous colonoscopies that were unrevealing. A biopsy of the colon taken near the larvae demonstrated eosinophilic infiltration. Microbiologic identification of collected samples showed saprophagous insect eggs that could not be speciated. Based on these findings, a diagnosis of intestinal myiasis was suggested. She was referred to an infectious disease specialist, and a bowel preparation regimen was initiated over multiple days to wash out the parasites, leading to the resolution of her symptoms.

## Discussion

The Centers for Disease Control and Prevention has linked intestinal myiasis to ingestion of fly-infested food, making it an uncommon manifestation in developed countries and more prevalent in areas with poor sanitation and tropical environments [1]. Synanthropic flies can deposit eggs on fruits or other foods that are then ingested, allowing larvae to pass through the gut and into the colon. Species diversity among implicated flies is broad, ranging from common houseflies (*Musca domestica*) and flesh flies (*Sarcophaga* spp.) to drain flies (*Clogmia albipunctata*), which thrive in indoor environments, such as drains and bathrooms, and deposit larvae that feed on decaying organic matter.

*C. albipunctata* has been documented in cases of intestinal myiasis, with a recent report emphasizing accidental ingestion of larvae-contaminated food, leading to symptoms such as nausea, vomiting, and abdominal discomfort, similar to those present in our patient [5]. Common symptoms of intestinal myiasis include abdominal pain, nausea, vomiting, diarrhea, and rectal itching, with our patient exhibiting intermittent abdominal pain and vomiting, likely reflecting episodic irritation by the larvae. However, not all cases present with symptoms; a prior case report described an asymptomatic Peruvian child with incidental findings of stool contaminated with larvae of *Sarcophaga* during examination [3]. Although some patients may be asymptomatic, other reports have noted abdominal discomfort and passage of live larvae in stool, contributing to variability in clinical presentation and delayed diagnosis [6]. Although the patient reported gastrointestinal symptoms for several years, it remains unclear whether intestinal myiasis was a longstanding process, or a more recent, intermittent exposure superimposed on chronic gastrointestinal complaints. This temporal uncertainty represents an important diagnostic challenge and limitation of this case. The transverse colonic stricture and wall thickening observed on imaging raised concern for chronic inflammatory changes; however, in the absence of follow-up endoscopic or radiologic evaluation, a direct causal relationship with larval presence cannot be definitively established.

Diagnosis of intestinal myiasis is often made by direct visualization of larvae in stool or during endoscopy, which was the key finding in our patient, who had live larvae on colonoscopy. To confirm the fly family, morphological identification of the larvae can be performed. It is important to differentiate true intestinal myiasis from pseudomyiasis, in which ingested larvae die in the gut without establishing infestation. True infestation requires larval survival and often implies ongoing presence or multiple larvae. The presence of numerous larvae in consecutive stool samples, or endoscopic findings with symptom relief after removal, supports true myiasis [7].

Management of intestinal myiasis is primarily mechanical and includes bowel lavage, washout, removal when accessible endoscopically, and measures to reduce re-exposure, such as improved food hygiene and sanitation in food preparation environments. Antiparasitic medications are not uniformly recommended or effective for many larvae, and evacuation/washout remains the first-line approach [8].

## Conclusions

This case highlights the diagnostic challenges posed by intestinal myiasis, a condition rarely encountered in developed regions, and often identified only when larvae are directly visualized. Our patient’s course illustrates how nonspecific symptoms and limited awareness can prolong diagnosis, with endoscopic detection providing diagnostic clarity. Recognition of potential exposure pathways, even years after migration, remains an important adjunct to diagnosis. Mechanical removal and hygiene counseling remain first-line management and the most effective treatment for intestinal myiasis. Increased clinician awareness is essential to avoid delayed diagnosis and ensure timely, appropriate care. As a single case report, this observation cannot be used to infer incidence, prevalence, or definitive risk factors, but rather serves to highlight a rare diagnostic consideration in patients with unexplained gastrointestinal symptoms. 
